# Bisoprolol and Bisoprolol-Valsartan Compatibility Studied by Differential Scanning Calorimetry, Nuclear Magnetic Resonance and X-Ray Powder Diffractometry

**DOI:** 10.1007/s11095-014-1471-7

**Published:** 2014-08-13

**Authors:** Marcin Skotnicki, Juan A. Aguilar, Marek Pyda, Paul Hodgkinson

**Affiliations:** 1 Department of Pharmaceutical Technology, Poznań University of Medical Sciences, ul. Grunwaldzka 6, 60-780 Poznań, Poland; 2 Department of Chemistry, Durham University, South Road, Durham, DH1 3LE UK; 3 Department of Chemistry, Rzeszów University of Technology, 35-959 Rzeszów, Poland

**Keywords:** bisoprolol, compatibility, DSC, SSNMR, valsartan

## Abstract

**Purpose:**

The objective of this study was to evaluate the thermal behavior of crystalline and amorphous bisoprolol fumarate and its compatibility with amorphous valsartan. This pharmacologically relevant drug combination is a potential candidate for fixed-dose combination formulation.

**Methods:**

DSC and TMDSC were used to examine thermal behavior of bisoprolol fumarate. SSNMR and XRPD were applied to probe the solid state forms. The thermal behavior of physical mixtures with different concentrations of bisoprolol and valsartan were examined by DSC and TMDSC, and the observed interactions were investigated by XRPD, solution- and solid-state NMR.

**Results:**

The phase transitions from thermal methods and solid-state NMR spectra of crystalline and amorphous bisoprolol fumarate are reported. Strong interactions between bisoprolol fumarate and valsartan were observed above 60 C, resulting in the formation of a new amorphous material. Solution- and solid-state NMR provided insight into the molecular nature of the incompatibility.

**Conclusions:**

A combined analysis of thermal methods, solution- and solid-state NMR and XRPD experiments allowed the investigation of the conformational and dynamic properties of bisoprolol fumarate. Since bisoprolol fumarate and valsartan react to form a new amorphous product, formulation of a fixed-dose combination would require separate reservoirs for bisoprolol and valsartan to prevent interactions. Similar problems might be expected with other excipients or APIs containing carboxylic groups.

**Electronic supplementary material:**

The online version of this article (doi:10.1007/s11095-014-1471-7) contains supplementary material, which is available to authorized users.

## Introduction

**Fig. 1:**
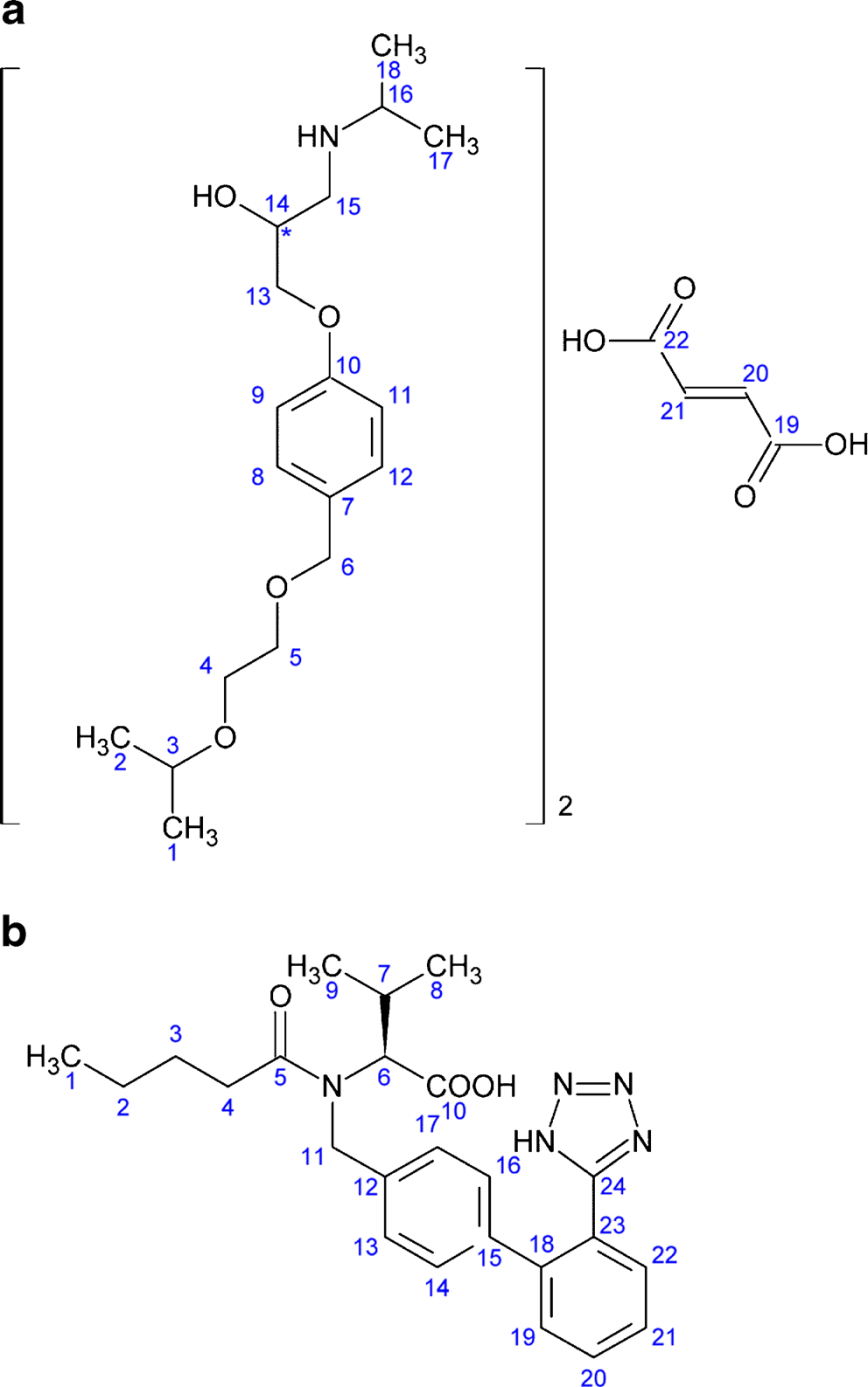
Chemical structures of (**a**) bisoprolol fumarate (*M* = 766.96 g mol^−1^) and (**b**) valsartan (*M* = 435.52 g mol^−1^) with the carbons numbered. The chemical structure of bisoprolol fumarate is that of the neutral molecules, as conventionally given. The material is, however, expected to be in a salt form as a solid, with the fumaric acid present as a doubly de-protonated fumarate ion and with the bisoprolol assumed to be protonated at its NH site.

Cardiovascular diseases (CVDs), such as coronary heart disease, heart failure, cerebrovascular disease or hypertension, are the major cause of death in developed countries (1). Bisoprolol fumarate (BISO), or (*RS*)-1-((alpha-(2-isopropoxyethoxy)-p-tolyl)oxy)-3-(isopropylamino)-2-propanol fumarate (2:1), Fig. 1a, is a beta_1_-selective (cardioselective) adrenoceptor blocking agent (2). Valsartan (VAL), or *N*-[*p*-(*o*-1*H*-tetrazol-5-ylphenyl)benzyl]-*N*-valeryl-L-valine, Fig. 1b, is a potent, highly specific angiotensin II type 1 (AT_1_) receptor antagonist (3). Both agents are used in the management of hypertension and heart failure (2, 3). Both are administered orally in a capsule or tablet form and may be used alone or in combination, but there is no single dosage form currently on the market containing both bisoprolol and valsartan. Combining drugs with synergistic mechanism of action in one dosage form (fixed-dose combination, FDC) has potential benefits, such as improved efficacy, reduced dosing, lower cost or enhanced patient compliance (4). Combination therapy has been shown to reduce CVD complications by more than 80% (4), and strong interest has been expressed by the pharmaceutical industry to develop an all-in-one pill (multicomponent cardiovascular pill, MCCP or polypill) containing an angiotensin-converting enzyme (ACE) inhibitor, beta-blocker, aspirin and statin (5). Both bisoprolol and valsartan (as an alternative for an ACE inhibitor) are good candidates for a two-ingredient FDC or MCCP.

The simplest and the most economic approach to formulating a multi-ingredient dosage form is a blend or granulation containing all the active pharmaceutical ingredients (APIs). The combination of different agents in a single dosage form can, however, lead to interactions between APIs, potentially affecting the stability and bioavailability of either component (6–12). Such interactions, termed incompatibilities, can be either physical or chemical in nature. Chemical interactions between APIs are well documented for FDCs intended for the treatment of tuberculosis, malaria or CVDs, conditions which usually require combination therapy (10–15). For example, ternary and quaternary drug combinations containing rifampicin and isoniazid along with pyrazinamide and/or ethambutol hydrochloride were very unstable, showing up to 70 or 95% loss of rifampicin or isoniazid, respectively, due to chemical reaction (11). Also, Kumar *et al.* reported incompatibility between polypill ingredients – atenolol, lisinopril, aspirin and statin due to chemical reactions (14, 15). Solid-state reactions of API with excipient or API can include: transacylation, Maillard browning reactions and acid–base reactions. As one of the most frequent approaches to improve physicochemical properties of an ionisable compound is to form a salt, and as formation of a free base/acid or new salt can significantly change physicochemical properties, acid–base reactions play an important role in formulation stability. Rohrs *et al.* reported decrease in dissolution rate for tablets of delavirdine mesylate following storage under accelerated stability conditions. This was found to result from formation of the less-soluble delavirdine free base due to acid–base reaction with excipient (croscarmellose sodium), as confirmed by Fourier transform infra-red spectroscopy (FT-IR) and solid-state NMR (SSNMR) (16). Zannou *et al.* found that maleate salt of a basic API showed a major loss in potency following stability testing, which again was attributed to conversion of the salt to the free base. The conversion strongly depended on microenviromental pH, and the formulation was successfully developed by acidification using citric acid as an excipient (17). Guerrieri and Taylor investigated by FT-IR and Raman spectroscopy four model pharmaceutical salts (two APIs mesylate and napsylate salts) mixed with common basic excipients and exposed to moderate relative humidity (18). They found that formation of free base depends on a number of factors such as pH_max_ of the salts (the pH of a solution where there is saturation of both ionized and unionized species), as well as the free base solubility. The conversion was also affected by excipient properties, including basicity, solubility, physical state and surface area.

Even in the absence of chemical reactions between components, the combination of two or more compounds (API-API or API-excipient) can result in physical interactions such as polymorphic transitions (19), amorphisation (20) or the formation of eutectic mixtures (21, 22). These can cause problems during manufacturing (22, 23), with shelf life (24) or bioavailability (25). For example, Bristol-Myers Squibb in 2010–11 recalled over 60 million tablets of an FDC product, Avalide®, containing an angiotensin receptor antagonist (irbesartan) and a diuretic (hydrochlorothiazide) due to the appearance of a less-soluble irbesartan polymorph (26). No such problems were observed with products containing irbesartan only. Zalac *et al.* have studied binary mixtures of antipyretic/analgesic drugs paracetamol and propyphenazone using differential scanning calorimetry (DSC), X-ray powder diffractometry (XRPD) and FT-IR spectroscopy (22). Studies revealed physical interactions related to eutectic formation and a lower degree of crystallinity of both components in the physical mixture, with the formation of eutectic mixture having a significant impact on the physical stability of the formulation. Rawlinson *et al.* studied interaction between cross-linked polyvinylpyrrolidone (PVP-CL) and the poorly soluble crystalline drug compound, ibuprofen, by DSC, XRPD, Raman and FT-IR spectroscopy (20). They reported a reduction in the crystallinity of ibuprofen observed by XRPD on simple physical mixing with PVP-CL, with amorphisation increasing during storage of the physical mixtures at ambient conditions.

Clearly the identification of possible interactions between APIs is extremely important at an early stage of drug development process. Thermal analysis is frequently used to study the physicochemical properties of APIs and compatibilities between drug-drug and drug-excipient (13, 22, 27, 28). DSC quickly provides information about possible interactions among the formulation components, according to the appearance, shift or disappearance of phase transition peaks and/or variations in the corresponding enthalpy or heat capacity values in thermal curves of drug-drug/excipient in physical mixtures (29, 30). However, the interpretation of the thermal data is not always trivial, and the interactions observed at high temperatures may not always be relevant to ambient conditions. Therefore, the use of complementary analytical techniques, such as XRPD or FT-IR, is advisable, as shown in research papers (31, 32) and as reviewed in Ref. (33). Solid-state nuclear magnetic resonance is another technique used to characterize pharmaceuticals (34, 35) and study physical or chemical interactions between APIs and excipients. It has been successfully used for characterizing the physical interactions occurring between API and polymers (36–39), between API and *β*-cyclodextrin (40), and acid–base reactions between API and excipients (16, 41, 42), but SSNMR has not been widely used, however, in API–API compatibility studies.

Bisoprolol belongs to class I of the Biopharmaceutics Classification System (BCS) (high solubility and high permeability) and is marketed in its crystalline form as a fumarate salt. Valsartan belongs to class II BCS (low solubility and high permeability) and is marketed in its amorphous form as a free acid. The aim of this study was to examine the thermal behavior of crystalline and amorphous bisoprolol fumarate and its compatibility with amorphous valsartan, employing thermogravimetric analysis (TGA), standard differential scanning calorimetry, temperature-modulated DSC (TMDSC), Fourier transform infra-red spectroscopy, solution- and solid-state nuclear magnetic resonance and powder X-ray diffractometry. The physicochemical properties of valsartan were reported by us elsewhere (43).

## Materials and Methods

### Materials

Pharmaceutical grade bisoprolol fumarate was obtained from Biofarm, Poznań, Poland and used without further treatment. The amorphous form of bisoprolol was prepared immediately prior to experimental measurements in the DSC pan in the instrument, in the NMR rotor in the probe, or on the hot stage of XRPD by heating sample to 120°C, holding for 5 min and then cooling with at least 10°C min^−1^ cooling rate to −50°C. Attempts to obtain diffraction-quality crystals by recrystallization from water, methanol, ethanol and ethyl acetate solutions were unsuccessful.

Valsartan (form AR, as-received, pharmaceutical grade) was obtained from Polpharma, Starogard Gdański, Poland and used without further treatment. Its fully amorphous form (form AM) was prepared immediately prior to experimental measurements in DSC pan in the instrument, in the NMR rotor in the probe, or on the hot stage of XRPD by heating sample to 140°C, holding for 20 min then cooling with at least approximately 3°C min^−1^ cooling rate to room temperature.

The purity of the provided materials was verified by solution ^1^H and ^13^C NMR. Physical mixtures of bisoprolol/valsartan in different concentrations from 10 to 95% (w/w) were prepared by mixing in a glass mortar for 20 min. Samples were used within 12 h. Melts were produced by heating of binary mixtures *in situ* in the DSC furnace, NMR probe or on the XRPD holder. The samples were melted at 120°C and cooled to −50°C with at least 10°C min^−1^ cooling rate.

## Thermogravimetric Analysis

TGA curves were obtained using a Mettler-Toledo TGA/DSC1 instrument or a Perkin Elmer Pyris 1 TGA instrument under a nitrogen gas flow 60 mL min^−1^. About 2 mg mass of powder samples were placed in an opened ceramic pan and heated at a rate of 10°C min^−1^ from 25 to 600°C.

## Differential Scanning Calorimetry

DSC curves were obtained using a DSC Q1000 TA Instrument Inc. (V9.9 Build 303) or a DSC 821 Mettler-Toledo instrument under a nitrogen gas flow of 50 and 60 mL min^−1^, respectively. Sample powders (1–10 mg) were crimped in a standard or hermetic aluminium pan and heated with different rates (1, 5 or 10°C min^−1^) from −50 to 140°C. Next, the samples were cooled down at 10, 20 or 50°C min^−1^ to room temperature and reheated to 140°C in a second run.

TMDSC curves were obtained using the DSC Q1000 TA Instrument Inc. instrument with an underlying heating rate 1°C min^−1^ and a temperature modulation with amplitude of 0.5°C and period of 60 s (which is sometimes termed “standard TMDSC”) from −50 to 140°C. The samples were then cooled at 10, 20 or 50°C min^−1^ to −50°C without modulation and reheated to 140°C in a second run.

The equipment was calibrated with indium (*m. p.* = 156.65°C, Δ*H*_f_ = 28.45 J g^−1^), and at least two tests were run on each sample. Melting, crystallisation and relaxation events are quoted as an onset temperature. Glass transition temperatures are quoted as midpoints. All values were determined using TA Universal Analysis 2000 V4.5A or Mettler-Toledo Star^e^ SW V10.0 software. Errors are quoted as one standard deviation.

## Nuclear Magnetic Resonance

Solution-state NMR experiments were carried out on a Varian VNMRS-700 machine with ^1^H and ^13^C frequencies of 699.73 and 175.97 MHz, respectively. Spectra of bisoprolol samples were measured in 0.5 mL of D_2_O and [D_6_]-DMSO. Spectra of valsartan and physical mixtures with bisoprolol were measured in 0.5 mL of [D_6_]-DMSO. All measurements were performed at 25°C. The diffusion ordered spectroscopy (DOSY) experiments were measured at 25°C on a 600 MHz Agilent spectrometer equipped with a probe with a *z*-gradient coil. The DBPPSTE (DOSY bipolar gradient pulses stimulated echo sequence) convection-compensated pulse sequence was used (44, 45) to acquire data sets in 31 min with 32 gradient amplitudes ranging from 5 to 45 G cm^−1^ in equal steps of gradient squared, using 32 transients, a total diffusion-encoding gradient duration of 2.0 ms, and a diffusion time of 200 ms.

Carbon-13 solid-state NMR spectra were generally recorded with cross-polarization (CP) and magic-angle spinning (MAS) using Varian VNMRS 400 and Varian Infinity Plus 500 spectrometers, operating at a ^13^C frequency of 100.56 and 125.68 MHz, respectively. Probes using 5 and 6 mm diameter rotors made of zirconia were employed. Typical operating conditions used a CP contact time of 2 ms, a recycle delay of 2 s, 512 to 2048 transients and spin rate of 10 kHz. Carbon chemical shifts were referenced to the signal for tetramethylsilane via a replacement sample of solid adamantane (*δ*_C_ = 38.4 ppm for the high-frequency line). Variable-temperature experiments were performed from −20 to 120°C, allowing samples to stabilize for 15–20 min before starting acquisition. All of the temperatures shown for ^13^C SSNMR experiments are quoted with a correction of +16°C above the displayed temperature, which is the estimated increase in sample temperature for a 5 mm rotor spinning at 10 kHz, based on previous calibration experiments using lead nitrate (46). While the VT measurements are reproducible (performed at least in duplicate), and relative temperatures are accurate to within ±1°C, the uncertainty on the absolute temperatures is estimated at ±4°C. The very different sample conditions in the different techniques used, in any case, limits the transferability of temperature scales. Spinning sidebands were identified with the aid of spectra acquired with 14 kHz spinning rate.

Proton spectra were recorded using Varian Infinity Plus 500 spectrometer operating at a ^1^H frequency of 499.70 MHz. A Bruker MAS probe using 1.3 mm diameter zirconia rotors was employed. Spectra were typically acquired using a recycle delay of 2 s, 4 to 64 transients and spin rates of 43 and 53 kHz. Data was also acquired at longer recycle delays (10 s) to verify that signals associated with slowly relaxing protons were not being missed. The estimated increase of the sample temperature with MAS rate of 53 kHz is estimated to be about 25°C; identical results (but with lower spectral resolution) were obtained at the lower spin rate. Proton broadline NMR spectra for static samples were measured at 400.17 MHz using a Bruker Avance III HD spectrometer. A MAS probe using 5 mm diameter rotors made of zirconia was employed. A recycle delay of 5 s was used and 32 transients were acquired. Spectra were measured over a temperatures range from 25 to 120°C, allowing at least 20 min for stabilization before acquisition.

Data were processed using gsim (47). A Gaussian line broadening of 40 Hz was applied to the ^13^C spectra and a “resolution enhancement” corresponding to an 80 Hz Gaussian function was applied to the ^1^H MAS spectra.

## Powder X-ray Diffractometry

X-ray diffraction patterns for most samples were obtained with a Bruker D8 ADVANCE instrument using a graphite bent-crystal monochromator (Cu K_α_). Samples were placed on a hot-stage sample holder and scanned in reflection mode from 10 to 40° 2*θ* over a temperatures range from 0 to 120°C. Samples were allowed to stabilize for 10–15 min before starting acquisition.

## Results

### Thermal Analysis

**Fig. 2:**
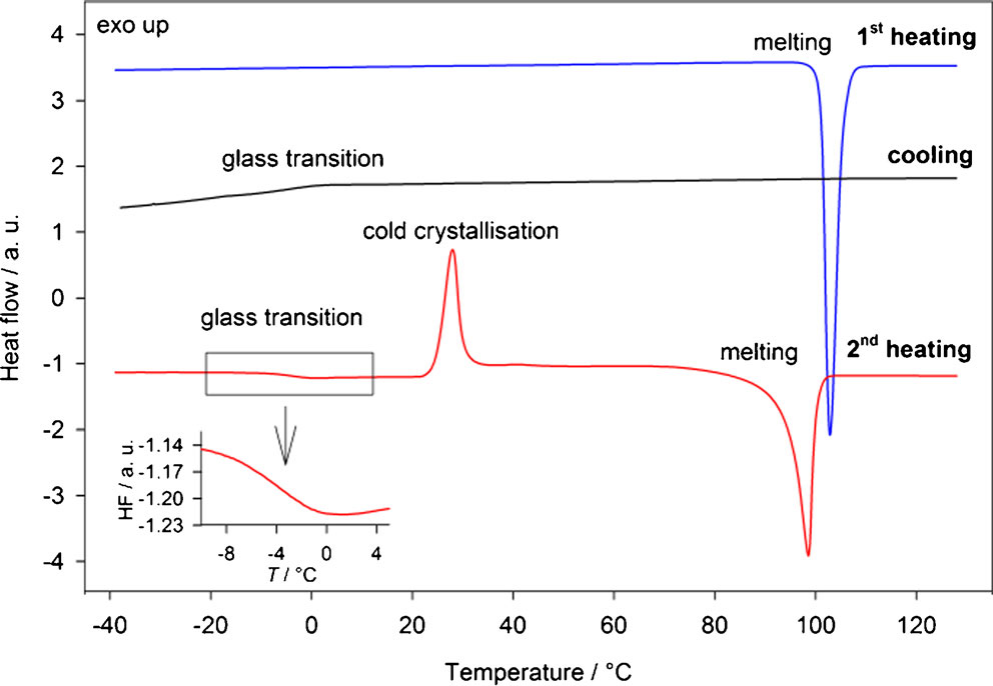
Standard DSC curves of crystalline bisoprolol showing 1st heating, cooling (glass transition of amorphised material) and 2nd heating (glass transition, cold crystallisation, melting of re-crystallised material). All runs obtained at a 10**°**C min^−1^ heating rate.

Figure 2 shows the standard DSC traces of crystalline bisoprolol with 10°C min^−1^ heating rate, cooling the sample to −50°C at 10°C min^−1^ and a second heating. On the first heating, the DSC curve shows one sharp endothermic peak with an onset at 102.3 ± 0.3°C and an enthalpy of fusion Δ*H*_BISO_ = 110 ± 2 J g^−1^ due to melting. Three events are observed on the second run: a glass transition at −3.6 ± 0.9°C with a change of heat capacity (Δ*C*_p_) of 0.51 ± 0.02 J g^−1^ K^−1^, followed by a cold crystallization exotherm at 24.2 ± 0.6°C (Δ*H* = 39 ± 2 J g^−1^) and a melting peak with onset at 95.4 ± 1.4°C (Δ*H* = 85 ± 1 J g^−1^). The lower values of the melting temperature and Δ*H* in the second heating may be due to differences in morphology in the re-crystallized phase of sample compared to the original crystalline material. There may be nanocrystalline domains present in the re-crystallized sample, which can lower the peak of melting, and/or the re-crystallization of the amorphous material may be incomplete. Both starting and re-crystallized material showed birefringence under polarized light microscopy, confirming their crystalline nature.

The previously reported (43) standard DSC curves of valsartan (form AR) show two endothermic events, one at around 60–90°C (Δ*H* = 5 ± 1 J g^−1^) corresponding to a loss of water / adsorbed solvent, and a second event with onset at 98.2 ± 0.9°C (Δ*H*_VAL_ = 26 ± 2 J g^−1^) corresponding to an enthalpy relaxation peak overlapped with a change of heat capacity. On the second heating run only one event is observed: a glass transition 73.9 ± 0.5°C and Δ*C*_p_ = 0.49 ± 0.01 J g^−1^ K^−1^.

**Fig. 3:**
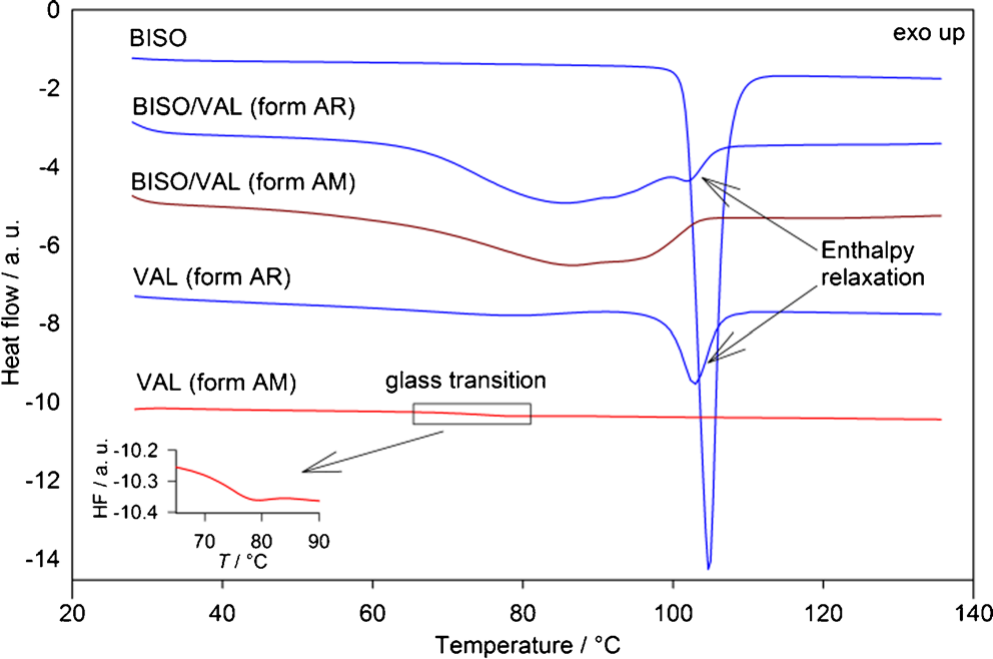
Standard DSC curves of the APIs and their 50/50 physical mixtures. The broad endothermic peak in the mixture indicates a significant physical or chemical interaction. All runs obtained at a 10**°**C min^−1^ heating rate

TGA curves (Figure 1S, supplementary material) of the individual components and a 50/50 (w/w) bisoprolol/valsartan (form AR) physical mixture show that the ingredients change individually in the mixture, indicating that no degradation due to interaction of the components takes place. However, the DSC curve of the same physical mixture, Fig. 3, does not show the expected sharp bisoprolol melting peak and instead shows overlapped broad endothermic peaks at around 60–100°C, indicating some physical or chemical interaction has disrupted the crystal lattice of bisoprolol. The total enthalpy of this peak is estimated to be 51 ± 5 J g^−1^, which is lower than the weighted sum of the enthalpies of the individual components, (Δ*H*_BISO_ + Δ*H*_VAL_)/2 ≈ 68 J g^−1^, suggesting interaction. Two minima at about 77 and 100°C are observed. The peak with minimum at around 100°C can be ascribed to the enthalpy relaxation peak of valsartan (form AR). This is confirmed by the DSC trace of a physical mixture made with freshly prepared amorphous valsartan (form AM), which shows only the broad peak with one minimum at 77°C, confirming that the peak at 100°C arises from valsartan (form AR). The interaction observed by DSC is also seen in a simple micro- and macroscopic observation; powder samples mixed and heated on the hot-stage to about 60°C convert into a more viscous state, and the material becomes sticky.

**Fig. 4:**
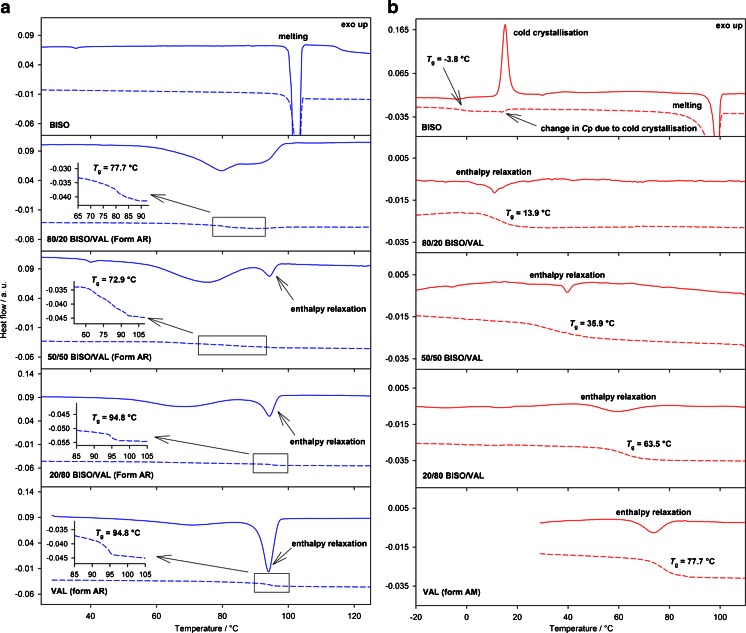
(**a**) 1st and (**b**) 2nd heating TMDSC experiments. Reversing (*dashed line*) signals show changes in glass transition and heat capacities. Non-reversing (*solid line*) signals show changes due to bisoprolol-valsartan interaction and enthalpy relaxation of valsartan (form AR), cold crystallization of bisoprolol, enthalpy relaxation of co-amorphous mixtures and valsartan (form AM).

TMDSC was employed to separate the kinetic and thermodynamic processes during heating for a selected number of samples. The total heat flow in a DSC experiment is the sum of the heat flow associated with the sample’s heat capacity and a second term associated with physical or chemical transformations (48, 49). By imposing a time-dependent modulation on the temperature ramp, time-dependent DSC decomposes the overall heat flow in a reversing and non-reversing heat-flow rate, corresponding to “thermodynamic” and kinetically limited processes, respectively. Figure 4a shows the reversing and non-reversing heat-flow rates obtained from TMDSC as a function of temperature for the pure components and physical mixtures in 80/20, 50/50 and 20/80 (w/w) ratios. TMDSC separates the relaxation enthalpy of valsartan, observed in the non-reversing signal, from the change in heat capacity at glass transition observed in the reversing signal. The non-reversing curves for the 20/80 and 50/50 physical mixtures (solid lines) clearly show the enthalpy relaxation peak ascribed to valsartan (form AR) and a broad endotherm most probably due to physical or chemical change of bisoprolol fumarate. The enthalpy relaxation in the mixtures is difficult to estimate precisely due to peak overlap, but the values are approximately 70 and 50% lower than expected (Δ*H*_50%VAL_ ~4 J g^−1^ compared to 50% Δ*H*_VAL_ = 13 J g^−1^, and Δ*H*_80%VAL_ ~11 J g^−1^ compared to 80% Δ*H*_VAL_ = 21 J g^−1^). It was not possible to estimate the value of enthalpy relaxation for the 80/20 mixture. The reversing curves of the physical mixtures, dashed lines in Fig. 4a, show only one event i.e. the glass transition. It is clear that there is a change in the glass transition temperature of valsartan in the 50/50 and 80/20 physical mixtures, and in its heat capacity in all mixtures. Standard DSC experiments performed for twelve physical mixtures from 0 to 100% of bisoprolol show the same behaviour (Figure 2S, supplementary material) as observed in TMDSC on the selected samples. In the physical mixtures with 90% bisoprolol by weight, the melting peak is broadened but can still be observed, while the melting peak was not identified with 80% or less bisoprolol in the mixture.

In the second heating of the physical mixtures, both components are amorphous. The TMDSC reversing curves of quench-cooled bisoprolol, Fig. 4b, show two thermodynamic processes: the glass transition and melting. As expected, the kinetically hindered cold crystallization process appears on the non-reversing curve. Neither melting nor cold crystallization is observed for co-melted 50/50 physical mixture; only a glass transition is observed. Compounds that are not miscible would show two separate glass transitions, one for each compound, while the co-melted bisoprolol/valsartan mixture only shows a single glass transition (*T*_g_ = 35.9°C) at a temperature intermediate between the *T*_g_ of pure drugs, with the temperature depending on the composition. These findings suggest that valsartan and bisoprolol had dissolved each other to form a homogenous amorphous mixture (50). Bisoprolol effectively lowers the *T*_g_ of valsartan, thus affecting its stability.

### NMR and XRPD Analyses

While DSC provides macroscopic information about the interaction between bisoprolol and valsartan, NMR and XRPD were used to probe the molecular basis for this behavior.

**Fig. 5:**
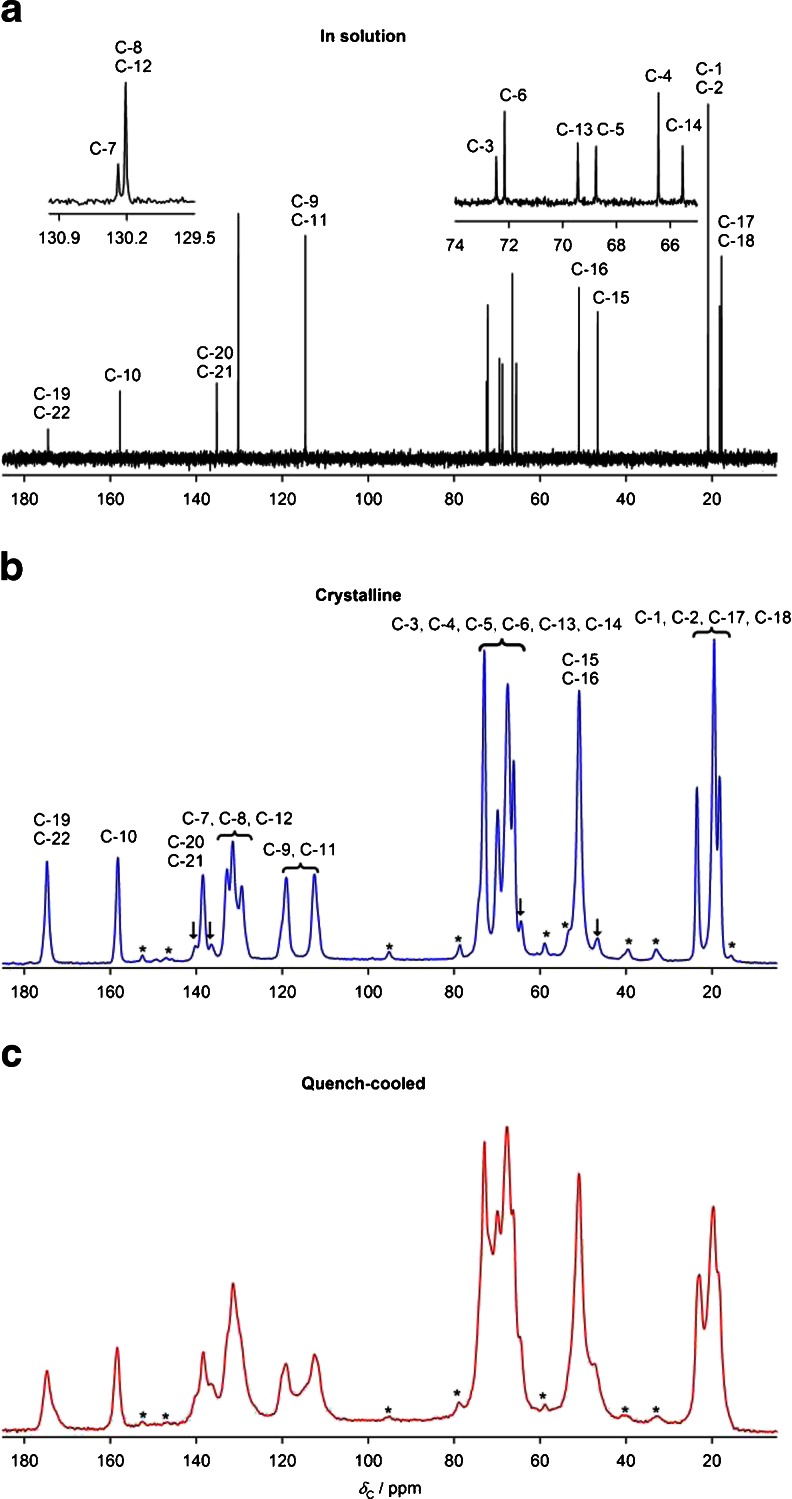
(**a**) ^13^C solution-state NMR spectrum of bisoprolol in D_2_O at 25**°**C, ^13^C CPMAS NMR spectra of (**b**) crystalline and (**c**) quench-cooled (amorphous) bisoprolol at −20**°**C. *Asterisks* (*) denote spinning sidebands. *Arrows* (↓) denote signals that are thought to arise from polymorphic or enantiomeric impurities.

Figure 5 shows the ^13^C solution-state spectrum of bisoprolol and ^13^C CP MAS NMR spectra of crystalline and quench-cooled (amorphous) bisoprolol. Due to the instability of the amorphous form at room temperature, spectra for both solid forms were acquired at −20°C i.e. below the glass transition temperature. Standard NMR techniques (COSY, HSQC and HMBC) were used to assign the solution-state spectrum. The solid-state spectrum was assigned using solution-state NMR data and spectral editing techniques: interrupted decoupling (dipolar dephasing) and depolarization experiments (inversion times from 25 to 100 μs). The resulting assignments are presented in Table I. The assignment of the solid-state spectra contains ambiguities due to its lower resolution and the differences in chemical shifts between the solution and solid state. Some of these ambiguities could, in principle, be resolved by computational prediction of chemical shifts given a crystal structure (51), but they are not significant for this study. Only one signal is observed for each site of bisoprolol and the two chemically distinct sites of the fumarate ion, indicating that the asymmetric unit cell contains a single bisoprolol molecule and a half molecule of fumarate. The spectrum of the crystalline material shows a set of low intensity signals, denoted by arrows in Fig. 5. Since there is no evidence from solution-state NMR of corresponding levels of chemical impurities, these signals could potentially arise from a second polymorphic form, or, more probably, enantiomeric “defects” (e.g. a *R*-molecule occupying a site otherwise occupied by *S* molecules). The low level of these signals (corresponding to about 1% of material) prevents further characterization. The spectrum of the quench-cooled form exhibits the expected general broadness of the resonances due to the range of local environments. These spectra vary slightly from experiment to experiment, presumably reflecting differences in cooling rates used to obtain the amorphous form. Standard NMR techniques were used to assign the solution-state spectrum of valsartan. The solid-state spectrum assignment was done based on solution-state data and by computational prediction of chemical shifts. Detailed solid-state NMR studies of pure valsartan will be published separately.

**Table I:** Solution- and solid-state NMR chemical shifts for bisoprolol and 50/50 (w/w) bisoprolol/valsartan physical mixture at 25°C (solution-state) and 38°C (solid-state).

Carbon No	Bisoprolol					Bisoprolol/valsartan (after heating to 80°C)	
	^1^H solution-state NMR / ppm (D_2_O)	^1^H solution-state NMR / ppm ([D_6_]-DMSO)	^13^C solution-state NMR / ppm (D_2_O)	^13^C solution-state NMR / ppm ([D_6_]-DMSO)	^13^C CPMAS NMR / ppm	^1^H solution-state NMR / ppm ([D_6_]-DMSO)	^13^C solution-state NMR / ppm ([D_6_]-DMSO)
17, 18	1.22 (dd, 12H)	1.12 (dd, 12H)	17.79, 18.21	20.48, 20.82	18.4, 19.9, 20.6, 23.3	1.21	18.61, 18.62, 19.19, 19.21^*a*^
1, 2	1.03 (d, 12H)	1.06 (d 12H)	20.93	22.49		1.06	22.48
15	3.10, 3.18 (2dd, 4H)	2.77, 2.92 (2dd, 4H)	46.63	48.65	51.2	2.93, 3.08	47.26, 47.28^*a*^
16	3.37 (sep, 2H)	3.04 (sep, 2H)	50.99	49.42		3.27	50.15
14	4.18 (m, 2H)	4.03 (m, 2H)	65.56	66.76	64.8 (CH_2_), 66.5 (CH), 67.8, 70.2 (CH_2_), 72.9, 73.6 (CH_2_)	4.12	65.57, 65.58^*a*^
4	3.51 (m, 4H)	3.46 (m, 4H)	66.45	67.18		3.47	67.20
5	3.51 (m, 4H)	3.46 (m, 4H)	68.79	69.57		3.47	69.60
13	3.95, 4.03 (2d, 4H)	3.92 (m, 4H)	69.46	70.76		3.93	70.29
6	4.39 (s, 4H)	4.38 (s, 4H)	72.19	72.11		4.38	72.09
3	3.58 (sep, 2H)	3.52 (sep, 2H)	72.49	71.27		3.52	71.28
9, 11	6.90 (d, 4H)	6.90 (d, 4H)	114.65	114.67	113.1; 119.2	6.88, 6.90^*a*^	114.38
8, 12	7.25 (d, 4H)	7.22 (d, 4H)	130.24	129.59	131.8, 133.1^*b*^	7.22	129.59
7	–	–	130.32	131.02	130.4	–	131.23
20, 21	6.37 (s, 2H)	6.39 (s, 2H)	135.26	136.07	138.6	6.53	135.16
10	–	–	157.79	158.34	158.7	–	158.13
19, 22	–	–	174.51	169.73	175.1	–	167.83

^*a*^ Resolution of signals most likely due to chiral centre and it is the subject of ongoing independent investigation. ^*b*^ At −20°C

**Fig. 6:**
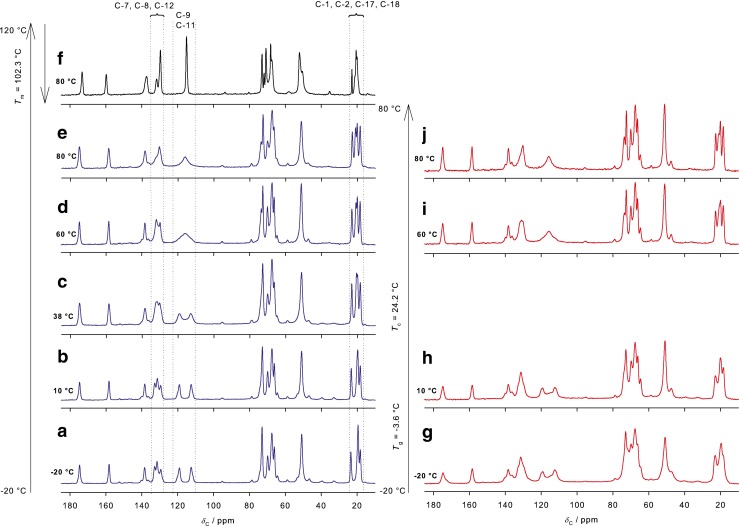
^13^C CPMAS NMR spectra of (**a**–**e**) crystalline, (**f**) melted then cooled to 80°C in the rubbery state and (**g**–**j**) quench-cooled bisoprolol as a function of calibrated temperature.

Figure 6a–e shows ^13^C CP MAS spectra of crystalline bisoprolol as a function of temperature. At low temperature, distinct signals are observed for the phenyl carbons (C-9 and C-11) at 113.0 and 119.3 ppm, indicating that the two halves of the phenyl ring are not related by symmetry in the crystal structure. The phenyl resonances coalescence (at 116.0 ppm) above 60°C, corresponding to increased molecular mobility of phenyl ring. This is commonly observed in molecular solids e.g. Ref. (52) and references therein. Carbon C-8 and C-12 exhibit similar behavior, although this is not clearly resolved due to overlap with the C-7 resonance. Subtle changes in the isopropyl signals (C-1, C-2, C-17 and C-18) are also observed, which are also consistent with increasing local mobility as the sample warms.

Figure 6f shows the ^13^C CP MAS NMR spectrum of bisoprolol heated above the melting point (to 120°C) and cooled to 80°C, above both the glass transition and re-crystallization temperatures. The much sharper spectrum is consistent with the sample being in a rubbery state, with a high degree of molecular mobility. As would be expected, the phenyl ring and isopropyl methyl carbon resonances are averaged in this state.

Figure 6g–j shows variable-temperature ^13^C spectra of quench-cooled bisoprolol. As discussed above, the spectrum of quench-cooled bisoprolol at −20°C is broad due to its glassy nature. At 10°C, above the glass transition but below the cold crystallization temperature, the spectrum is slightly more resolved and sharper than at −20°C, consistent with increased mobility and hence motional averaging in the rubbery state. The appearance of relatively narrow signals at 60 and 80°C shows that the bisoprolol has re-crystallized (35).

**Fig. 7:**
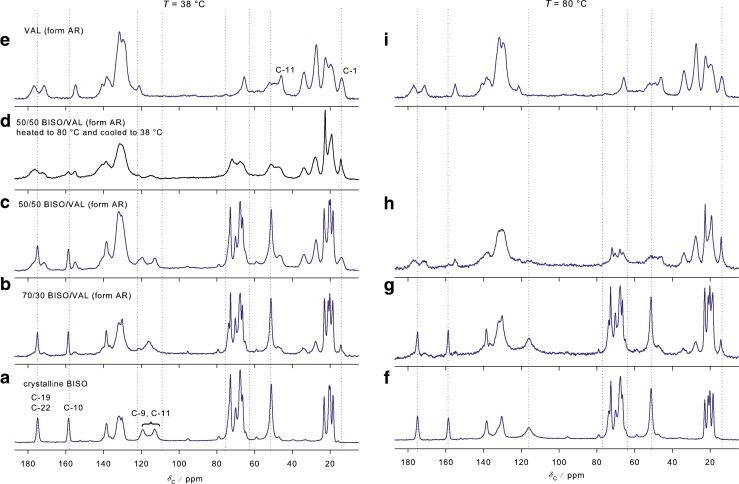
^13^C CPMAS NMR spectra of each API and physical mixtures in different concentrations at 38 and 80°C.

Figure 7 shows the ^13^C NMR spectra of bisoprolol, valsartan and 70/30 and 50/50 physical mixtures at 38 and 80°C. The spectra at 38°C correspond to appropriately weighted combinations of the spectra of the pure drugs, showing no evidence of interactions between the components at ambient temperature. There are changes in the aromatic region (C-9, C-11) for the 70% bisoprolol/valsartan mixture, implying increased molecular mobility of phenyl ring. This observation is puzzling, and may simply reflect a strong sensitivity of this spectral feature to sample temperature. The spectrum of the 50/50 (w/w) physical mixture changes significantly at 80°C, Fig. 7h, with the peaks associated with the bisoprolol having much lower intensities. There are also some subtle changes in the valsartan resonances, e.g. some sharpening of the methyl carbon (C-1), suggesting an increase in mobility, and a slight decrease in intensity of C-11, but these are difficult to interpret with confidence. In contrast to the DSC results, there are no significant changes observed at 80°C for the 70/30 and 80/20 physical mixtures in comparison to the pure drugs (data not shown).

**Fig. 8:**
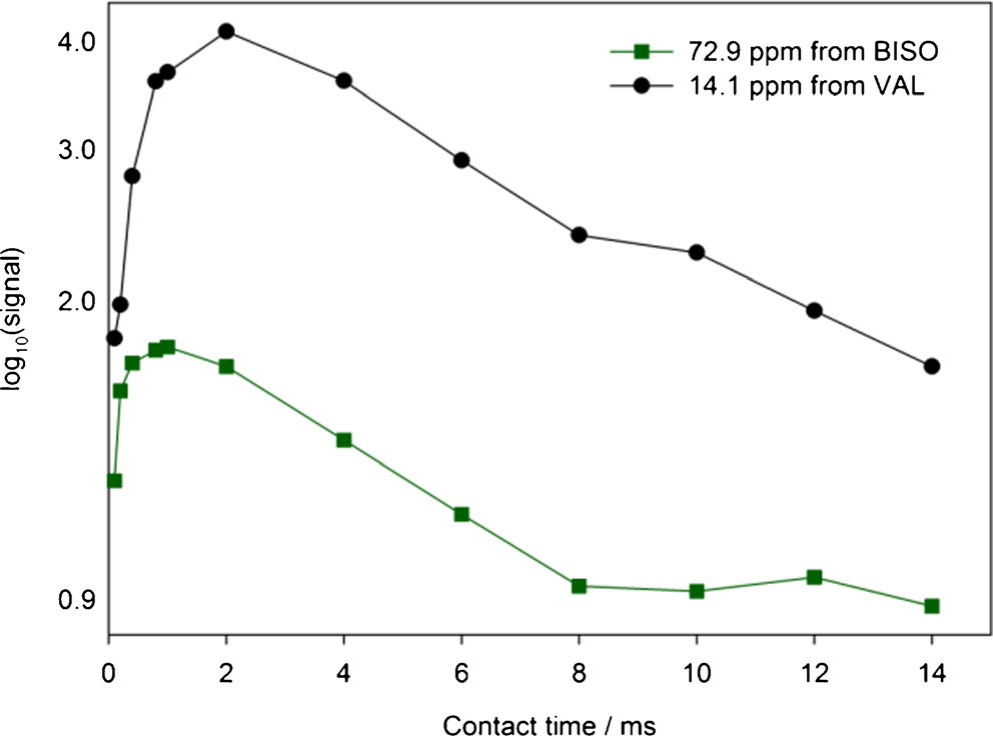
^13^C NMR signal intensity vs. cross-polarization contact time for the CH_3_ at 14.1 ppm of valsartan and the alkyl signal at 72.9 ppm of bisoprolol in their 50/50 (w/w) physical mixture at 80°C.

Figure 7d shows the spectrum of the 50/50 (w/w) physical mixture of bisoprolol/valsartan heated to 80°C and then cooled to 38°C. The spectrum at 38°C is similar to the spectrum at 80°C, i.e. the reduction in intensities of the peaks associated with crystalline bisoprolol is irreversible. Experiments using longer recycle delays (up to 16 s) confirmed that the loss of these peaks was not an artifact of relaxation times – components with long spin–lattice relaxation would be suppressed in experiments with short relaxation delays between scans. The loss of bisoprolol signals could also be associated with a dramatic increase in molecular mobility, which would selectively reduce the efficiency of cross-polarization for mobile components. However, direct excitation of the ^13^C spectrum, using recycle delays of up to 50 s, did not reveal signals from mobile components that could be assigned to bisoprolol. To investigate the apparent disappearance of the bisoprolol signals in the 50/50 physical mixture spectrum, the intensities of the methyl (C-1, 14.1 ppm) resonance of valsartan and the alkyl signal at 72.9 ppm of bisoprolol in the binary mixture were measured as a function of the cross-polarization contact time, Fig. 8. The slope of decaying part of the curve, which corresponds to *T*_1ρ_ relaxation, is similar for both carbons. This is consistent with them being mixed at a molecular level or in domains smaller than 5 nm (34) and also shows that the weakness of the bisoprolol signals is not due to rapid *T*_1ρ_ relaxation during cross-polarization. The bisoprolol signals are present, but just very weak and broad, presumably as a result of the distribution of local environments and hence dispersion of chemical shifts for each resonance due to amorphisation.

**Fig. 9:**
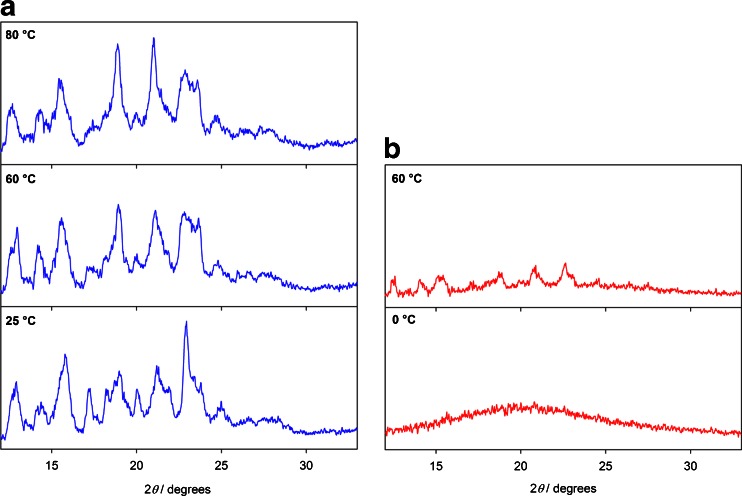
Variable-temperature X-ray diffractograms of (**a**) crystalline and (**b**) quench-cooled bisoprolol.

Figure 9 shows the X-ray diffraction patterns of crystalline and quench-cooled bisoprolol as a function of temperature. The diffraction patterns of the crystalline material at 60 and 80°C show only modest changes of peak intensity and no significant changes in peak position, suggesting that there is no significant structural change during heating, which agrees with the DSC and NMR findings. XRPD of the quench-cooled bisoprolol at 0°C, i.e. below the re-crystallization temperature, shows a characteristic halo pattern confirming its amorphous nature. At 60°C, the material has re-crystallized into the same form as the starting material. However, the diffraction peaks are much weaker, suggesting a lower degree of crystallinity. This is consistent with the lower melting point of bisoprolol in the second heating and the slightly broader and less-resolved NMR resonances of quench-cooled bisoprolol at 60°C, Fig. 6i.

**Fig. 10:**
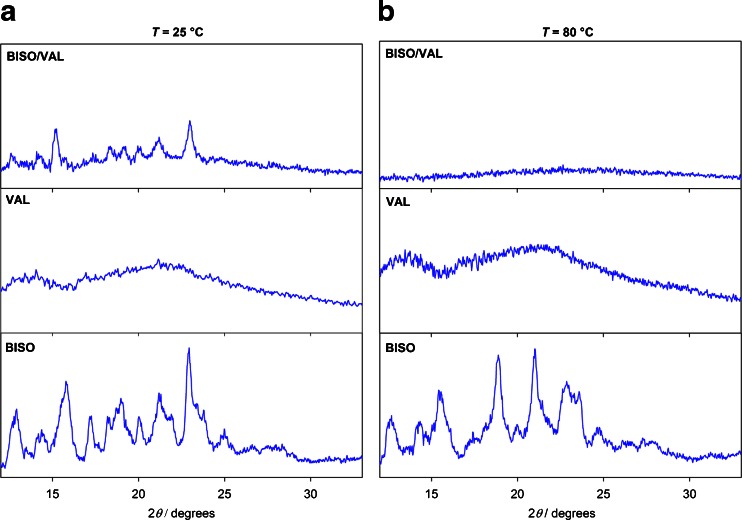
X-ray diffractograms of crystalline bisoprolol, amorphous valsartan (form AR) and a 50/50 (w/w) physical mixture of bisoprolol/valsartan (form AR) at (**a**) 25°C and (**b**) 80°C.

XRPD patterns of the 50/50 physical mixture, Fig. 10, show peaks corresponding to bisoprolol at 25°C. Although some studies suggest that the lack of observed interactions via XRPD at ambient temperature proves that DSC is a more sensitive technique (27, 53), variable-temperature XRPD experiments are necessary in order to meaningfully compare the two methods. A decrease of signal intensity from the crystalline bisoprolol component starts at about 60°C (data not shown), and at 80°C the XRPD pattern implies that the material is fully amorphous. In the second heating, the amorphous halo observed from 0 to 60°C confirms that the material remains amorphous (data not shown), as found by other methods. In contrast to the solid-state NMR results on 70/30 and 80/20 mixtures, but in agreement with DSC, XRPD measurements on the 70/30 physical mixture showed only partial amorphisation of bisoprolol, with some of bisoprolol remaining in its crystalline state (Figure 3S, supplementary material).

**Fig. 11:**
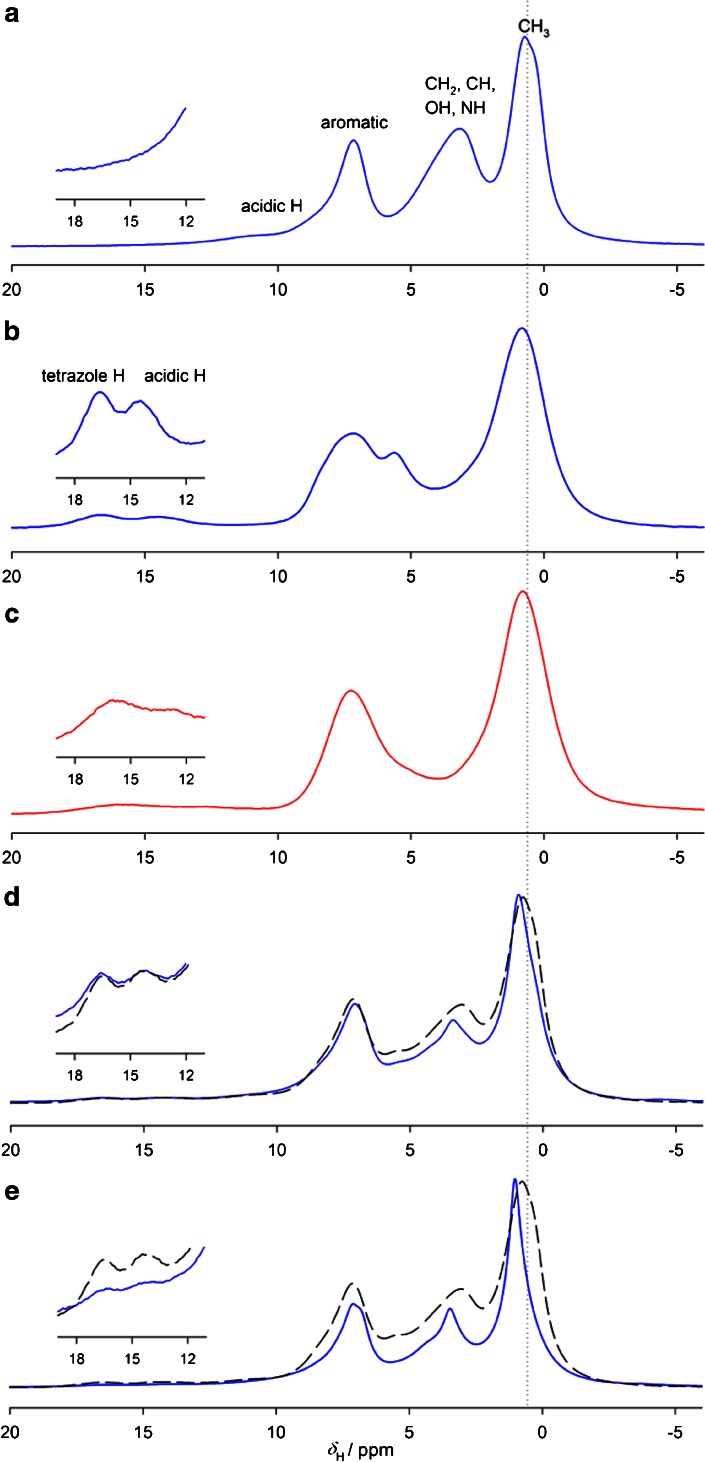
^1^H MAS NMR spectra of (**a**) bisoprolol, (**b**) valsartan (form AR), (**c**) valsartan (form AM), (**d**) 50/50 physical mixture of bisoprolol/valsartan (solid line) (**e**) 50/50 physical mixture of bisoprolol/valsartan heated to 80°C (*solid line*). (**d**) and (**e**) show also the sum spectra of valsartan and bisoprolol (*dashed line*). Spectra were recorded at an MAS rate of 53 kHz. Sharper resonances in physical mixtures indicate increased mobility (amorphisation) of the mixture (**d**–**e**).

To investigate the potential involvement of the COOH and NH groups in the interaction between the drugs, ^1^H MAS NMR studies were also performed using very fast magic-angle spinning (53 kHz) to reduce the line-broadening associated with the strong dipolar interactions between ^1^H spins (54). Four different resonances are distinguishable in the fast MAS ^1^H spectrum of bisoprolol, Fig. 11a, corresponding to methyl (≈0.7 ppm), methylene, methine, hydroxyl, amine (2–5.5 ppm), aromatic (≈7 ppm) protons and also a low intensity signal at ≈ 10.4 ppm corresponding to an acid proton bonded to amine group. Figure 11b and c show spectra of the two valsartan forms, clearly showing differences in the hydrogen bonding region at 12–18 ppm. Figure 11d shows spectra of physical mixture of both APIs (solid line) and the sum of spectra of the pure materials (dashed line) The lines of the physical mixture are slightly sharper than the sum spectrum, confirming increased mobility at the onset of transition; increasing the spinning rate of the sample of untreated physical mixture to 63 kHz resulted in the sample volume expanding, presumably as the increased frictional heating triggered the interaction at about 60–80°C. This volume expansion is consistent with the crystalline bisoprolol transforming into an amorphous form with lower density. Figure 11e shows the spectra of the physical mixture heated in an oven to 80°C and cooled to room temperature. The lines are now significantly sharper than the spectrum of the pure materials, due to increased molecular mobility in the amorphised material. The spectrum also shows changes in the hydrogen bonding region i.e. in acidic and tetrazole protons signals arising from valsartan, which implies a change of hydrogen bonding due to amorphisation and/or deprotonation of these acidic carbons. The differences in overall mobility were confirmed by static ^1^H NMR; the bandshape of untreated physical mixture is measurably broader (Δ*υ*_½_ = 37.6 kHz) then that from the mixture heated to 80–85°C and recorded at room temperature (Δ*υ*_½_ = 28.9 kHz), consistent with increased motion in the amorphised material (Figure 4S, supplementary material).

To further investigate the nature of the interactions, 1D and 2D solution-state NMR experiments were performed. Although there is a report related to degradation of bisoprolol in acidic environments (55), spectra of untreated and heated to 80°C bisoprolol/valsartan physical mixtures do not show significant differences, indicating no significant degradation after heating the sample to the interaction temperature (80°C) and holding the mixture at this temperature for 10–15 min. Comparing the spectra of bisoprolol fumarate in isolation and in its physical mixture reveals some significant differences in ^1^H and ^13^C chemical shifts, Table I. These differences are largest for nuclei close to the amine group. For example, the methyl carbons (C-17, C-18) show increased shifts of 0.09 ppm for ^1^H and 1.6–1.9 ppm for ^13^C, while the methylene carbon (C-15) shift is 1.4 ppm lower in the mixture with valsartan. The fumarate carboxylic carbons (C-22, C-19) shifts also change significantly (Δ*δ*_C_ = −1.9 ppm), implying a change in association of the fumarate ions. Comparing the valsartan spectra is more difficult due to the presence of two conformers in solution associated with hindered rotation of its amide bond, see Refs. (56, 57). Changes are seen for the carbonyl group (C-5) and in the aromatic region (Table IS, supplementary material). These are associated with changes of conformational distribution of valsartan due to interaction with bisoprolol, and are not discussed further in this paper. There is also a noticeable change in shift of the carboxylic carbon (C-10, Δ*δ*_C_ = −0.55 and −0.61 ppm for the major and minor conformer, respectively). Similar information was provided by FT-IR spectroscopy (Figure 5S, supplementary material).

More direct evidence of interaction between valsartan and bisoprolol fumarate is provided by the diffusion ordered spectroscopy (DOSY) experiments, Figure 6S of the supplementary material. In D_2_O solution, the fumarate species has a significantly faster diffusion rate (by a factor of 1.5) than the bisoprolol, as would be expected from its much smaller molecular mass and size, Figure 6S(A). In DMSO solution, however, the diffusion rates of fumarate and bisoprolol are similar (*D*_F_ = 1.746 (±0.006) × 10^−10^ m^2^ s^−1^ and *D*_B_ = 1.865 (±0.005) × 10^−10^ m^2^ s^−1^), implying that they diffuse as an ion pair; this pairing is disrupted in D_2_O solution, which allows the fumarate to diffuse more freely, Figure 6S(B). The presence of valsartan has a similar effect in the DMSO solution, Figure 6S(C); the valsartan and bisoprolol species migrate at the similar rate (*D*_B_ = 1.52 (±0.01) × 10^−10^ m^2^ s^−1^ and *D*_VAL_ = 1.552 (±0.007) × 10^−10^ m^2^ s^−1^), with the fumarate diffusing 23% faster than bisoprolol. This strongly suggests that the valsartan and bisoprolol are preferentially associating, leaving the fumarate species to diffuse more quickly. Although the behaviour of molecules in solution cannot be directly compared to their behaviour in the solid state, these observations are consistent with the disruptive effects of valsartan on the crystalline bisoprolol fumarate.

## Discussion

Bisoprolol fumarate is found to have glass transition below room temperature (−3.6°C) and to be unstable in amorphous form, undergoing re-crystallization upon heating to the same polymorphic form as starting material but probably with a lower degree of crystallinity.

Knowing the glass-forming ability of APIs from their crystalline state is very important for development of the solid dosage form. Amorphous solid forms usually show improved solubility. The solubility improvement of amorphous form is not relevant to bisoprolol as it is freely soluble, but formation of amorphous API might increase the rate of chemical degradation and cause problems during manufacturing (42, 58). Baird *et al.* (59) divided the crystallization tendency of drug into 3 classes. Drugs showing only a crystallization peak (*T*_c_) and no glass transition (*T*_g_) signal during the 1st cooling of melted drug belong to class 1, those that do not undergo crystallization during the first cooling but become crystalline at above *T*_g_ during second heating, to class 2 and those that do not convert into crystalline form during cooling/heating/heating cycle, to class 3. Bisoprolol fumarate is found to belong to class 2. The crystalline form of valsartan could not be obtained, but the thermal behavior of its amorphous form indicates that it belongs to class 3.

Compatibility studies of crystalline bisoprolol fumarate and two amorphous forms of valsartan (free acid) were completed over a range of temperatures from room temperature to above the phase transitions of both APIs. DSC and TMDSC revealed the interaction of the APIs by the appearance of a new peak and the disappearance of the bisoprolol melting peak in the physical mixtures. Such lowering or shifting of the melting peak in mixtures might occur as a result of the formation of a eutectic mixture, which is a common phenomenon known for APIs (60). However, eutectics are generally considered in terms of initially crystalline components, whereas in this case one of the materials is amorphous, having a *T*_g_ rather than a melting point. Moreover, the TMDSC reversing curve does not show an endothermic melting peak, suggesting no eutectic formation has occurred. Instead the new transition peak appears as a kinetic signal, confirming irreversibility of the process and indicating chemical or physical interaction. The ^13^C CP MAS NMR and XRPD measurements clearly show the interactions as the samples are heated to 80°C. The ^13^C NMR spectrum of the 50/50 physical mixture contained only broad and low intensity signals, suggesting that the bisoprolol has interacted with valsartan and amorphised. This is consistent with the picture from XRPD. The static ^1^H bandshapes suggest increased overall mobility in the amorphised material, while the ^1^H spectra obtained at ultra-fast MAS rates showed significant changes in the signals in the hydrogen bonding region arising from the acidic COOH and tetrazole hydrogens of valsartan.

A likely cause of the interaction is the competition between valsartan and fumarate for bisoprolol amine group. Valsartan has two acidic centres, the COOH group (p*K*_a1_ = 3.6) and the tetrazole ring (p*K*_a2_ = 4.7) (61), while bisoprolol is a salt of bicarboxylic fumarate acid (p*K*_a1_ = 3.0, p*K*_a2_ = 4.4) (62). There is a possibility of H^+^ exchange as valsartan has a lower p*K*_a1_ than p*K*_a2_ for fumaric acid. It is worth noting that valsartan as a free acid is successfully formulated with besylate salt API (Exforge®, amlodipine besylate), but benzenesulfonic acid is a much stronger acid (p*K*_a_ = 0.70) than valsartan, and so acid–base reaction is unlikely.

Although p*K*_a_ values describe equilibrium phenomena in solution, it is widely accepted that they can be a valuable tool for predicting acid–base interactions in solid-state (63). It is possible that the dominant interaction in the solid state could be protonation of the doubly deprotonated fumarate in bisoprolol fumarate by the acidic proton of valsartan, corresponding to a 1:1 molar ratio of APIs. The mole ratios in the investigated 20/80, 50/50, 70/30 and 80/20 (w/w) bisoprolol/valsartan physical mixtures are as follows: 1:7.04, 1:1.76, 1:0.76, 1:0.44. ^13^C CP MAS NMR measurement of 50/50 mixture revealed interaction, whereas the 70/30 and 80/20 spectra showed clear resonances from bisoprolol suggesting that a significant fraction of the bisoprolol fumarate was largely unchanged. The XRPD measurements indicated amorphisation of the mixture at 50/50 concentration, and experiments with the 70/30 mixture showed an increased halo pattern, confirming some amorphisation, but also some unaltered bisoprolol fumarate diffraction peaks. In the 50/50 mixture there is enough valsartan to displace one fumarate carboxylate, whereas in the 70/30 and 80/20 mixtures there is excess of bisoprolol fumarate, and thus crystalline bisoprolol can still be detected by NMR and XRPD. The TMDSC results show similar behavior with concentration; the non-reversing (kinetic) heat flow shows an enthalpy relaxation peak corresponding to valsartan AR form in the 20/80 and 50/50 BISO/VAL physical mixtures but not in the 80/20 mixture, where all valsartan had “reacted”. The DSC shows evidence of the interaction over a wide range of concentrations; the melting peak is broadened but is observed in the physical mixtures with 90% bisoprolol concentration, while the melting peak was not identified with 80% or less of bisoprolol in the physical mixture. The results show that the addition of small amounts of valsartan (>10%) leads to major changes in the solid state of bisoprolol fumarate. In this respect, the DSC is more sensitive than XRPD and NMR, which both struggle to observe low fractions of amorphous content.

## Conclusions

The crystalline and amorphous forms of bisoprolol and its compatibility with two amorphous forms of valsartan were analyzed. Amorphous bisoprolol has low stability and re-crystallizes above the glass transition. The compatibility study has shown that simple blending of the APIs to produce a fixed-dose formulation of bisoprolol and valsartan is unsuitable due to physical and chemical reaction which causes amorphisation into a new bisoprolol/valsartan material at elevated temperatures, and potentially also under long-term storage. Thus, formulation of an FDC or polypill containing bisoprolol and valsartan would require physical separation of the ingredients to ensure a stable product. Similar problems might be expected with excipents or APIs containing carboxylic group; for example with aspirin or folic acid. It was demonstrated that thermal methods play a pivotal role in early detection of API-API interaction leading to incompatibilities. Solution- and solid-state NMR and XRPD provide information about the molecular nature of these interactions. Variable-temperature NMR and XRPD experiments are seen to be an ideal complement to thermal methods in the investigation of drug-drug incompatibilities as the kinetics of potential interactions may be too slow to be detected at ambient temperature.

## Electronic supplementary material

Below is the link to the electronic supplementary material.

ESM 1 (DOCX 667 kb)

## Abbreviations

ACE: Angiotensin-converting enzyme
API: Active pharmaceutical ingredient
BCS: Biopharmaceutics classification system
BISO: Bisoprolol fumarate
COSY: Correlated spectroscopy
CP: Cross-polarization
CVD: Cardiovascular disease
DBPPSTE: DOSY bipolar gradient pulses stimulated echo sequence
DE: Direct excitation
DOSY: Diffusion ordered spectroscopy
DSC: Differential scanning calorimetry
FDC: Fixed-dose combination
FT-IR: Fourier transform infra-red spectroscopy
HMBC: Heteronuclear multiple bond correlation
HSQC: Heteronuclear single quantum correlation
MAS: Magic-angle spinning
MCCP: Multicomponent cardiovascular pill
PVP-CL: Cross-linked polyvinylpyrrolidone
SSNMR: Solid-state nuclear magnetic resonance
TGA: Thermogravimetric analysis
TMDSC: Temperature-modulated DSC
VAL: Valsartan
XRPD: X-ray powder diffractometry
