# Supplementary material for: Bisoprolol and Bisoprolol-Valsartan Compatibility Studied by Differential Scanning Calorimetry, Nuclear Magnetic Resonance and X-Ray Powder Diffractometry
Source: Pharm Res. 2014 Aug 13;32(2):414–29. doi: 10.1007/s11095-014-1471-7 (PMC4300422; doi:10.1007/s11095-014-1471-7)
Supplement: Supplementary file 1 — (DOCX 667 kb) [file 11095_2014_1471_MOESM1_ESM.docx]

**Supplementary material**

**Bisoprolol and bisoprolol-valsartan compatibility studied by Differential Scanning Calorimetry, Nuclear Magnetic Resonance and X-ray Powder Diffractometry**

Marcin Skotnicki^1,2^_,_ Juan A. Aguilar^2^, Marek Pyda^3^ and Paul Hodgkinson^2^

^1^Department of Pharmaceutical Technology, Poznań University of Medical Sciences, ul. Grunwaldzka 6, 60-780 Poznań, Poland

^2^Department of Chemistry, Durham University, South Road, Durham DH1 3LE, United Kingdom

^3^Department of Chemistry, Rzeszów University of Technology, 35-959 Rzeszów, Poland

**Figures**


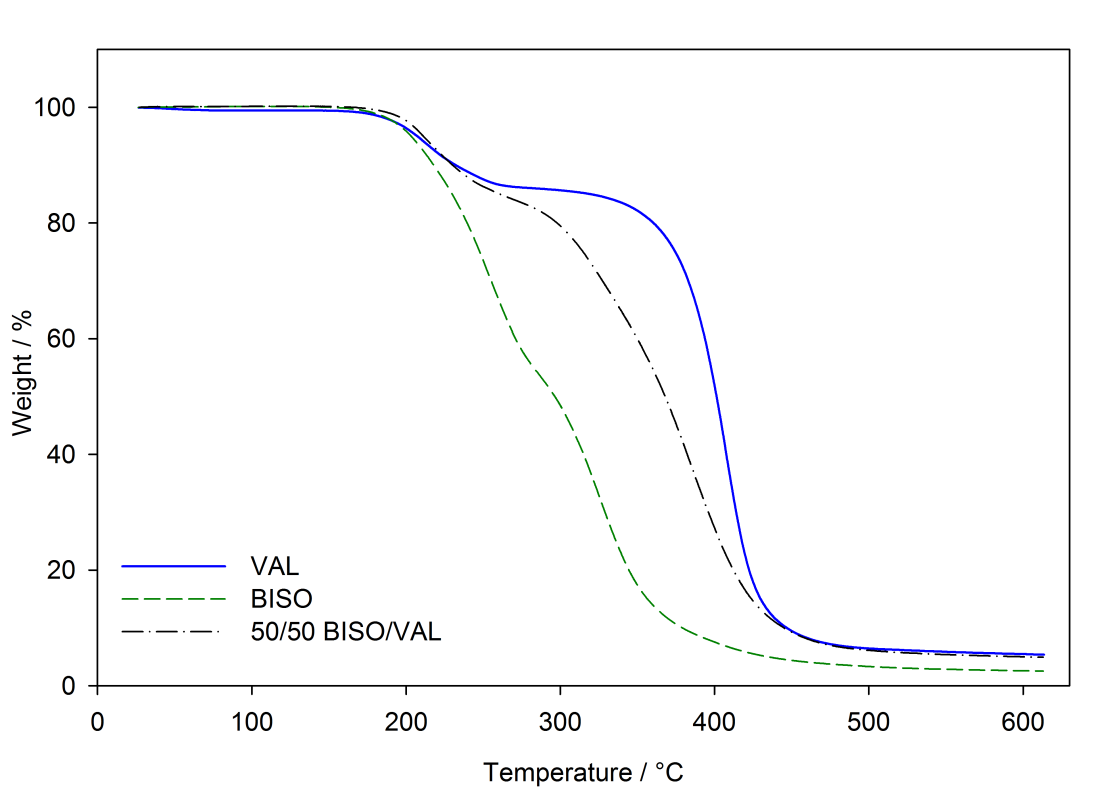


Figure 1S. TGA curves for valsartan (form AR), crystalline bisoprolol and 50/50 (w/w) physical mixture of bisoprolol/valsartan (form AR), showing no evidence of mass loss before the degradation points of the individual components.


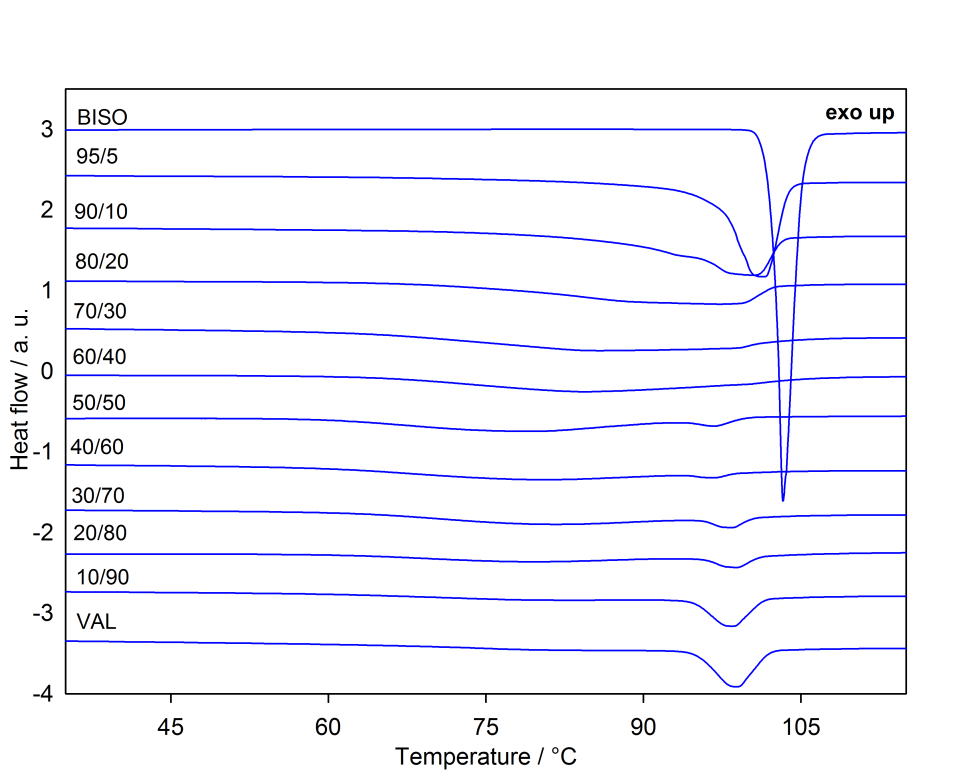


Figure 2S. Standard DSC curves obtained at a 5 °C min^–1^ heating rate for APIs and their physical mixtures in a various weight ratios.


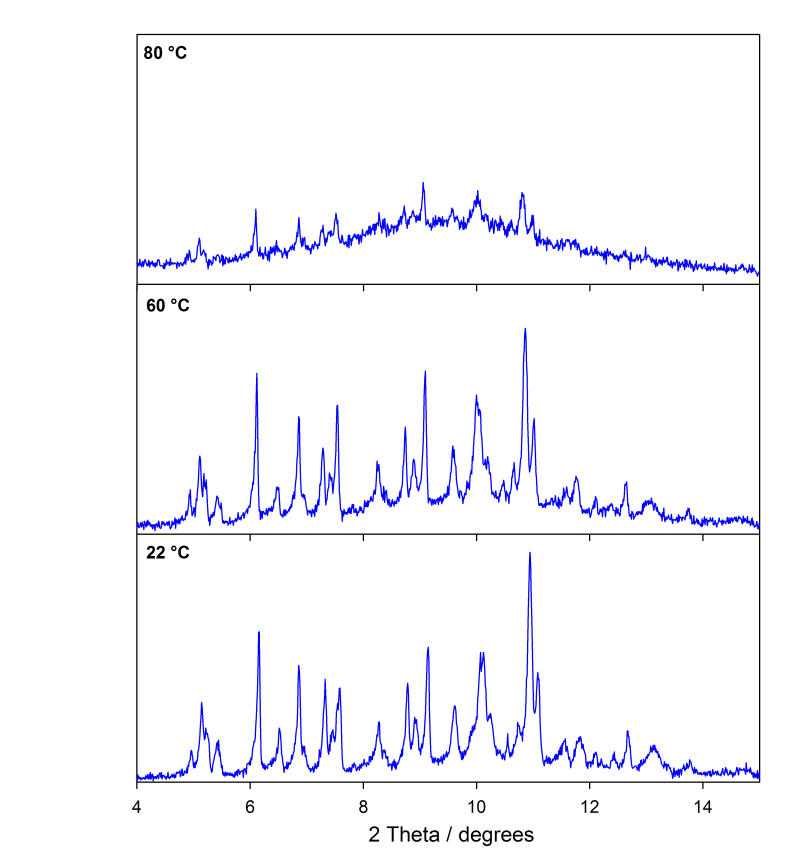


Figure 3S. X-ray diffractograms of a 70/30 (w/w) bisoprolol/valsartan (form AR) physical mixture at 22, 60 and 80 °C. The results obtained at 80 °C clearly show only partial amorphisation of bisoprolol, with some of bisoprolol remaining in its crystalline state. Please note that the results are obtained from diffractometer operating at Mo K_α_ radiation in contrast to diffractograms shown on Figures 9 and 10 in the main text which were obtained using Cu K_α_ radiation.


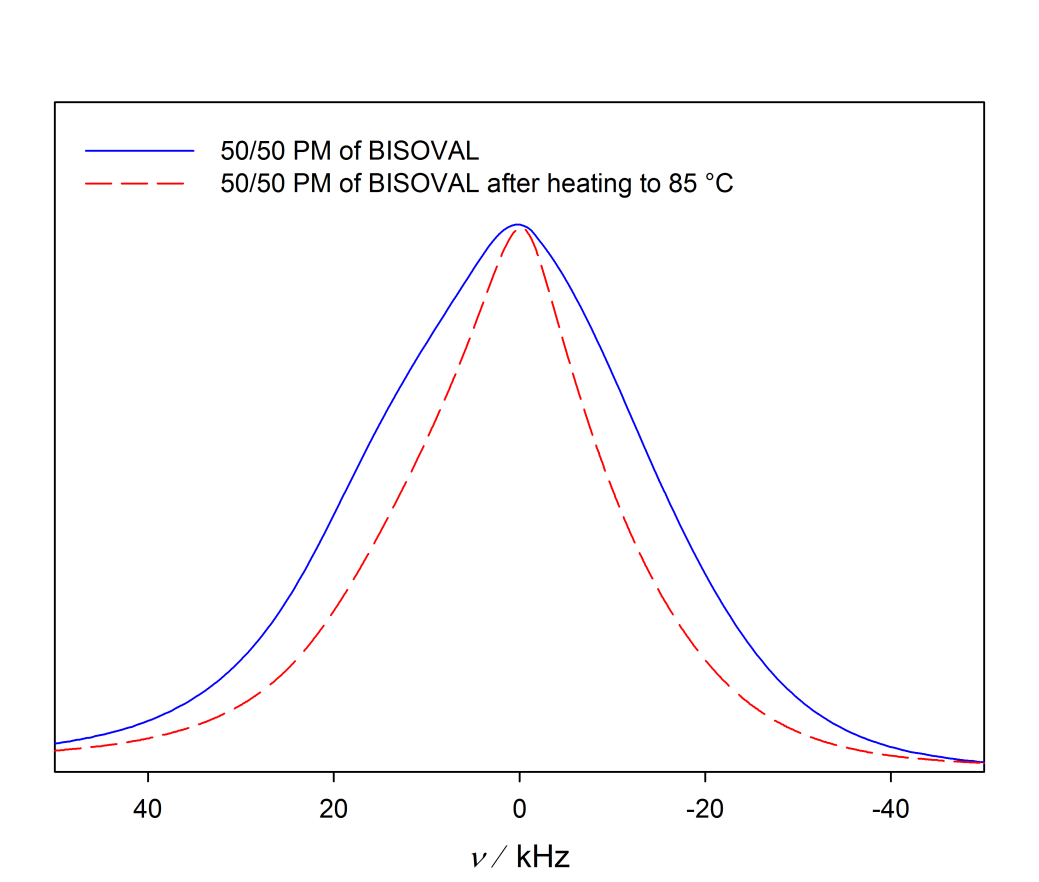


Figure 4S. Proton static spectra of untreated (solid line) and heated to 85 °C (dashed line) 50/50 (w/w) bisoprolol/valsartan physical mixtures recorded at 25 °C. The bandshape of untreated physical mixture is measurably broader (Δ*υ*_½_ = 37.6 kHz) then that from the mixture heated to 85 °C (Δ*υ*_½_ = 28.9 kHz), implying increased motion in the amorphised material.


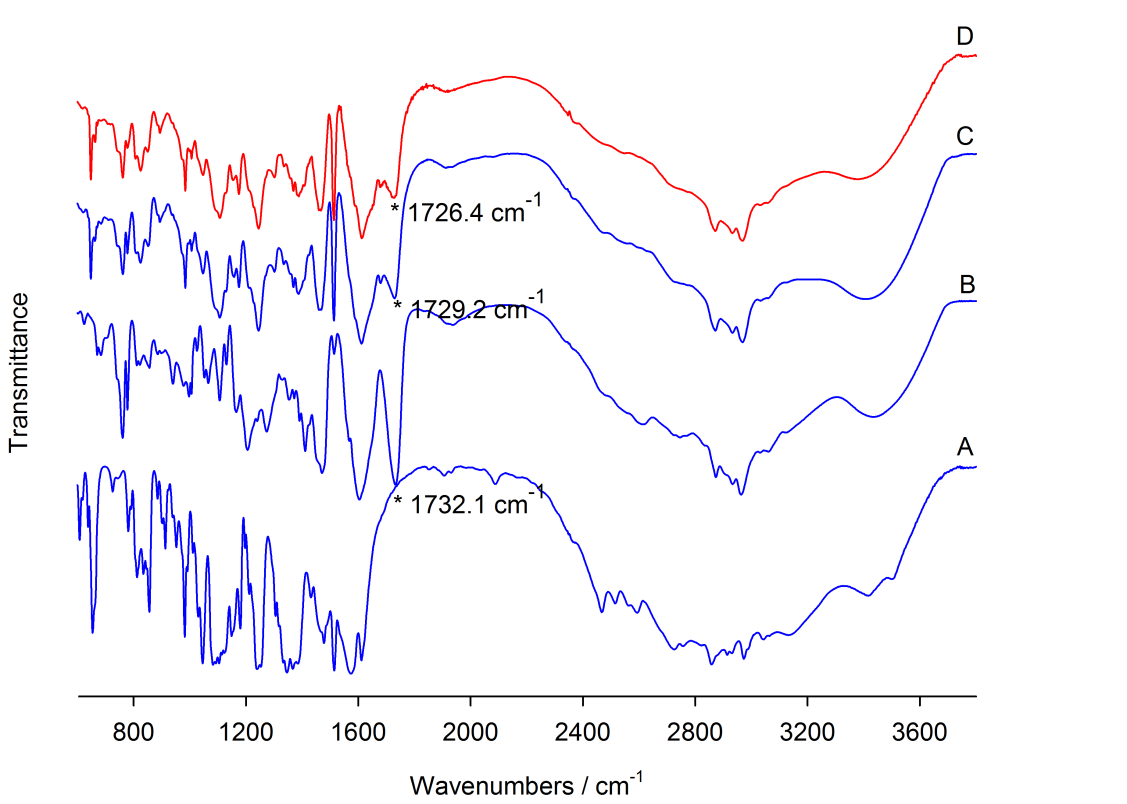


Figure 5S. FT-IR spectra of (A) bisoprolol, (B) valsartan (form AR), (C) untreated and (D) heated to 80 °C 50/50 (w/w) bisoprolol/valsartan physical mixtures acquired at room temperature. Spectra show significant shift of the carbonyl group band of valsartan (C-10, *υ*_C=O_ = 1732.1 cm^–1^), indicating the change in the local environment of carboxylic group. Due to peak overlap, it was not possible to obtain specific information about the interaction of bisoprolol in the physical mixture.

A
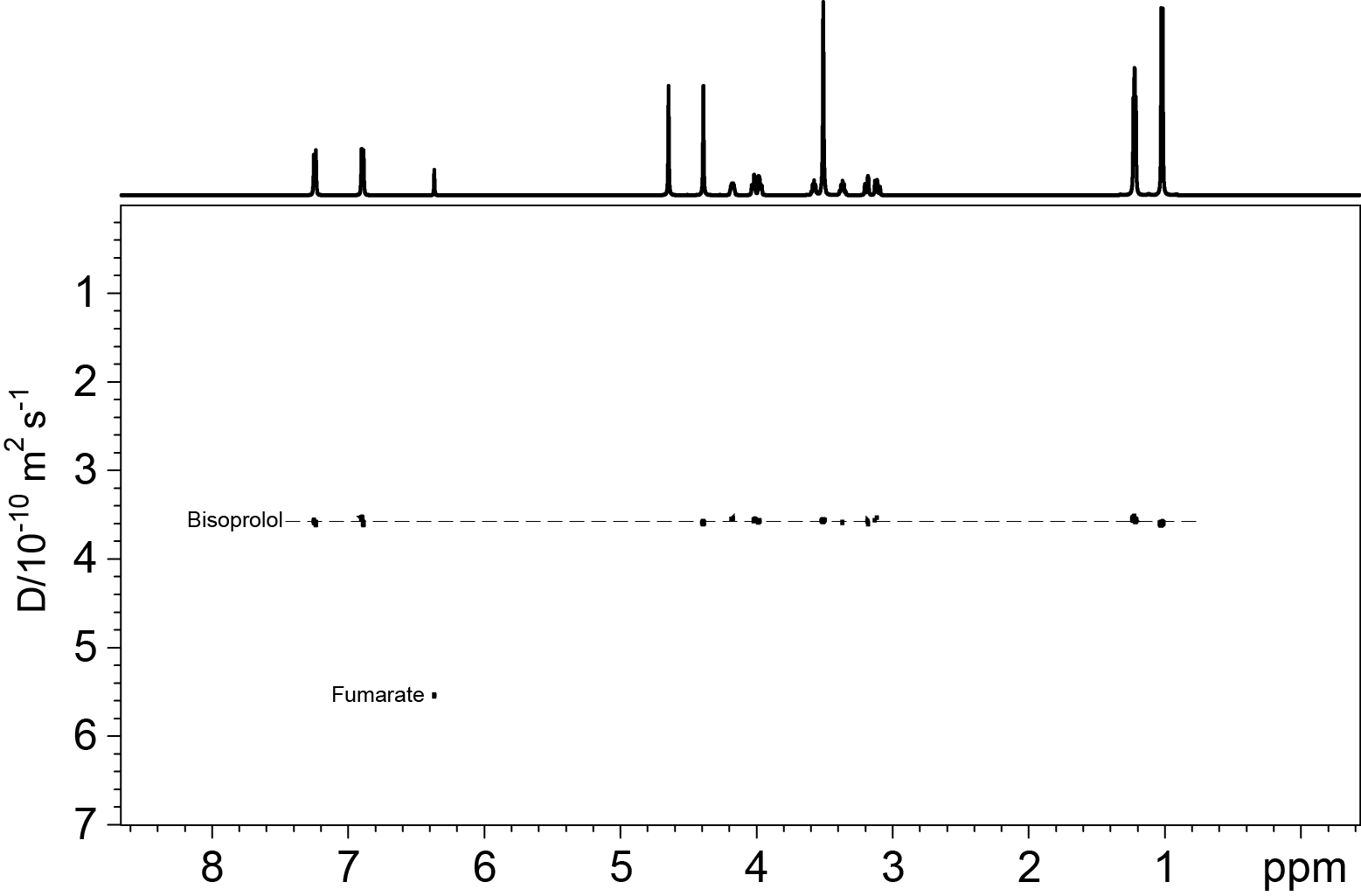


B
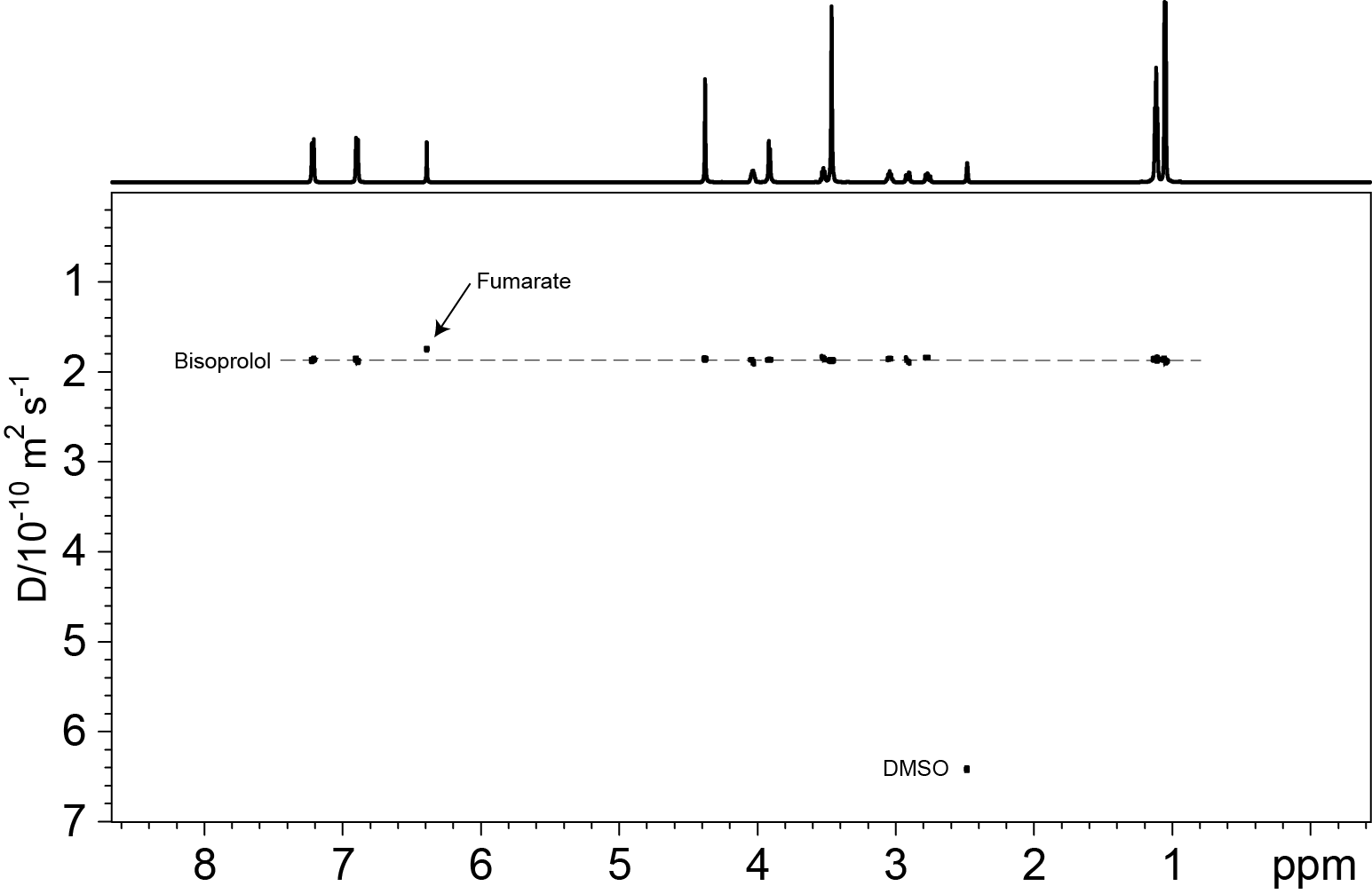


C**
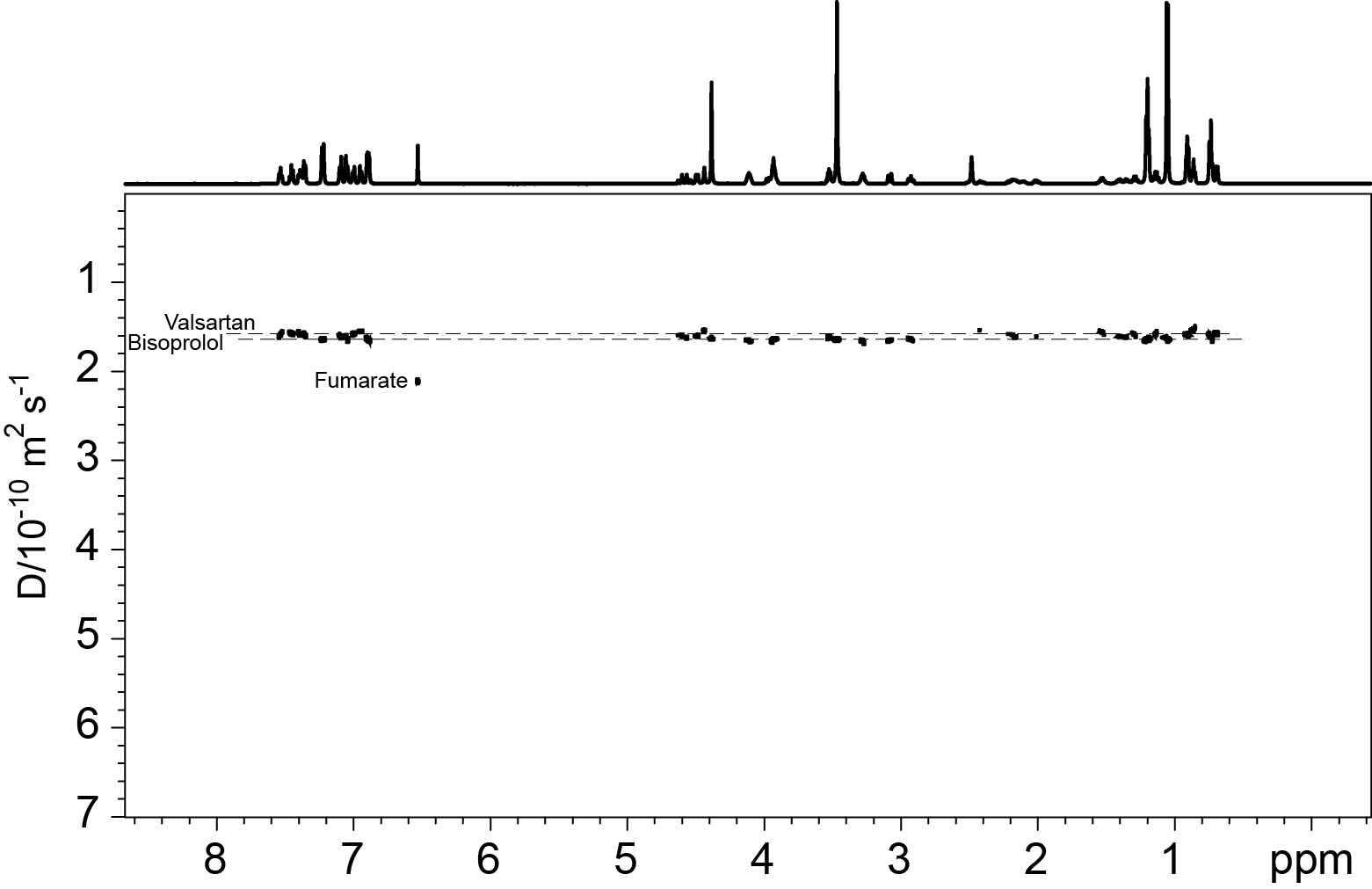
**

Figure 6S. 2D-DOSY spectra of (A) bisoprolol fumarate in D_2_O, (B) bisoprolol fumarate in [D_6_]-DMSO and (C) 50/50 (w/w) physical mixture of bisoprolol/valsartan in [D_6_]-DMSO. The diffusion coefficient of fumarate increases in presence of valsartan, suggesting competition of valsartan with fumarate.

**Table**

Table IS. Solution-state NMR chemical shifts for valsartan and 50/50 (w/w) bisoprolol/valsartan physical mixture at 25 °C in [D_6_]-DMSO. M and m denote major and minor conformers, respectively.

| **Carbon No** | **Valsartan** | | | | **Bisoprolol/valsartan (after heating to 80 °C )** | | | |
| --- | --- | --- | --- | --- | --- | --- | --- | --- |
|  | **^1^H shift / ppm** | | **^13^C shift / ppm** | | **^1^H shift / ppm** | | **^13^C shift / ppm** | |
|  | **M** | **m** | **M** | **m** | **M** | **m** | **M** | **m** |
| **1** | 0.74 | 0.87 | 14.16 | 14.25 | 0.73 | 0.86 | 14.16 | 14.27 |
| **2** | 1.13 | 1.30 | 22.12 | 2.27 | 1.14 | 1.29 | 22.14 | 22.33 |
| **3** | 1.39 | 1.53 | 27.26 | 27.45 | 1.41 | 1.53 | 27.34 | 27.52 |
| **4** | 2.00, 2.18*^a^* | 2.45, 2.50*^a^* | 32.90 | 32.94 | 2.00, 2.18*^a^* | 2.41, 2.50*^a^* | 32.96 | 33.07 |
| **5** | - | - | 173.91 | 173.88 | - | - | 173.89 | 173.74 |
| **6** | 4.445 | 4.07 | 63.39 | 66.18 | 4.50 | 3.97 | 63.49 | 67.31 |
| **7** | 2.18 | 2.12 | 28.00 | 28.04 | 2.18 | 2.10 | 28.08 | 28.21 |
| **8** | 0.74 | 0.69 | 18.25 | 18.93 | 0.73 | 0.685 | 19.23 | 19.11 |
| **9** | 0.92 | 0.92 | 20.59 | 19.84 | 0.91 | 0.91 | 20.56 | 20.06 |
| **10** | - | - | 172.34 | 172.07 | - | - | 172.89 | 172.68 |
| **11** | 4.61 | 4.46 | 49.15 | 45.90*^b^* | 4.56, 4.61*^a^* | 4.45, 4.46*^a^* | 48.81 | 45.90 |
| **12** | - | - | 138.22 | 137.55*^b^* | - | - | 137.39 | 138.12 |
| **13** | 7.19 | 7.08 | 126.72 | 127.37 | 7.10 | 7.02 | 126.14 | 126.89 |
| **14** | 7.05 | 6.96 | 129.24 | 128.16 | 7.05 | 6.95 | 129.46 | 128.89 |
| **15** | - | - | 138.22 | 138.67 | - | - | 138.82 | 139.67 |
| **16** | 7.05 | 6.96 | 129.24 | 128.16 | 7.05 | 6.95 | 129.46 | 128.89 |
| **17** | 7.19 | 7.08 | 126.72 | 127.13 | 7.10 | 7.02 | 126.14 | 126.89 |
| **18** | - | - | 141.65 | 141.78 | - | - | 141.01 | 141.27 |
| **19** | 7.51 | 7.51 | 130.98 | 131.05 | 7.37, 7.41, 7.47, 7.55 | | 129.16, 129.46 130.68, 131.04 | |
| **20** | 7.62 | 7.62 | 130.98 | 131.07 |  |  |  |  |
| **21** | 7.55 | 7.55 | 128.14 | 128.01 |  |  |  |  |
| **22** | 7.67 | 7.67 | 131.51 | 131.51 |  |  |  |  |
| **23** | - | - | 123.84*^c^* | 123.84*^c^* | - | - | Not detected | Not detected |
| **24** | - | - | Not detected | Not detected | - | - | Not detected | Not detected |

*^a^* Chemical shift of nonequivalent protons of methylenes. *^b^* Low and *^c^* very low intensity peaks. Low intensities will reflect long ^13^C relaxation times of quaternary sites, but peaks may also be broadened by motional exchange.
